# Decoupling the Lattice Distortion and Charge Doping Effects on the Phase Transition Behavior of VO_2_ by Titanium (Ti^4+^) Doping

**DOI:** 10.1038/srep09328

**Published:** 2015-05-07

**Authors:** Yanfei Wu, Lele Fan, Qinghua Liu, Shi Chen, Weifeng Huang, Feihu Chen, Guangming Liao, Chongwen Zou, Ziyu Wu

**Affiliations:** 1 State Key Laboratory for Mesoscopic Physics, Department of Physics, Peking University, Beijing. 100871, People's Republic of China; 2 National Synchrotron Radiation Laboratory, University of Science and Technology of China, Hefei. 230029, People's Republic of China; 3 Institute of High Energy Physics, Chinese Academy of Science, Beijing. 100049, People's Republic of China

## Abstract

The mechanism for regulating the critical temperature (T_C_) of metal-insulator transition (MIT) in ions-doped VO_2_ systems is still a matter of debate, in particular, the unclear roles of lattice distortion and charge doping effects. To rule out the charge doping effect on the regulation of T_C_, we investigated Ti^4+^-doped VO_2_ (Ti_x_V_1-x_O_2_) system. It was observed that the T_C_ of Ti_x_V_1-x_O_2_ samples first slightly decreased and then increased with increasing Ti concentration. X-ray absorption fine structure (XAFS) spectroscopy was used to explore the electronic states and local lattice structures around both Ti and V atoms in Ti_x_V_1-x_O_2_ samples. Our results revealed the local structure evolution from the initial anatase to the rutile-like structure around the Ti dopants. Furthermore, the host monoclinic VO_2_ lattice, specifically, the VO_6_ octahedra would be subtly distorted by Ti doping. The distortion of VO_6_ octahedra and the variation of T_C_ showed almost the similar trend, confirming the direct effect of local structural perturbations on the phase transition behavior. By comparing other ion-doping systems, we point out that the charge doping is more effective than the lattice distortion in modulating the MIT behavior of VO_2_ materials.

Vanadium dioxide (VO_2_), a strongly correlated electron and metal-insulator transition (MIT) material, is an extremely interesting material suitable for many technological applications. The most striking features of VO_2_ are its abrupt first-order MIT near 68° C, exhibiting a large change in the resistivity (up to five orders of magnitude) and the infrared transmittance/reflectivity in the sub-picoscecond time scale^1^. Simultaneously, the crystal structure transforms from a high-temperature tetragonal rutile (R) structure characterized by a single V−V distance of 2.85 Å and linear chains of edge-shared Jahn−Teller-distorted VO_6_ octahedra, to a low-temperature monoclinic (M1) structure containing V^4+^–V^4+^ pairs forming a zigzag chain with alternating V−V distances of 2.65 and 3.12 Å and more distorted VO_6_ octahedra (see Figure 1). Moreover, different external stimuli such as thermal, electrical, optical, or magnetic field can trigger the MIT in VO_2_^2^. These unique characteristics make VO_2_ suitable for application such as smart windows^3^, memory devices^4^, uncooled microbolometers^5^, electronic/optical switch devices^6^,^7^, thermal/chemical sensors^8^,^9^, etc.

The typical MIT temperature of the pure VO_2_ is ~68°C, which unfortunately is not ideal for practical applications. Much effort has been devoted to regulate the MIT critical temperature (T_C_) of VO_2_. An effective route for regulating the T_C_ is doping with metal ions^10^,^11^,^12^, in addition to adding internal/external stress^13^,^14^ or controlling the microstructure and defects^15^,^16^,^17^. In the available literatures, it was shown that the T_C_ could be decreased by doping large dopants ions with higher-valence such as W^6+^, Mo^5+^ and Nb^5+^, or increased by small dopants ions with lower-valence (Al^3+^, Cr^3+^, and Ga^3+^)^12^,^18^,^19^,^20^,^21^,^22^,^23^. In this framework, the still open challenge is the deeper understanding of the intrinsic mechanism for the regulation of T_C_ by ions-doping, which have an important significance in VO_2_ based functional devices.

The intuitive understanding is that the regulation of the T_C_ by metal doping inside the VO_2_ lattice depends on the relative size and the relative valence of the dopant ion compared to that of the V^4+^ ion^22^,^23^. The substitution may give rise to the changes of lattice structure and carrier density (or the conductivity) in parent VO_2_^11^,^19^,^24^,^25^. In the case of the W^6+^-doping, it has been considered that the W^6+^-doping can donor two extra-electrons to the VO_2_ host if considering the charge neutralization. The increase of the electron density affects the band structure and the activation energy, facilitating the transformation to the metallic phase^25^,^26^. Moreover, each W dopant is doped substitutionally and disrupts the dimeric V^4+^–V^4+^ bond to form W^6+^–V^3+^ and V^4+^–V^3+^ pairs. This replacement destabilizes the monoclinic phase and thus lowers the energy barrier for the transition to the rutile structure^26^,^27^,^28^. The different contributions finally result in the reduction of T_C_. Other researchers focused on the influence of local structure perturbations induced by dopant ions^23^,^28^. They suggested that the T_C_ was not affected by the carrier density variation in VO_2_, but by the lattice distortion induced by the dopants with different ion radius. The change trend of T_C_ can be correlated with the relative size of the dopant ion compared to that of the V^4+^ ion. Booth et al^28^. claimed that the effect of W^6+^ dopants on neighboring cells would be only structural. Based on EXAFS data, they concluded that a local rutile structure was formed around W^6+^ dopants and a significant expansion in the [110]_R_ and 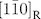 directions induced by W^6+^ dopants broke the dimeric homopolar V-V pairs due to the decreasing d_∥_ orbital overlap, showing the lattice deformations towards the high-temperature rutile structure, and thus resulting in the reduction of T_C_. Recently, a combination of XANES and EXAFS spectra has been used to characterize the electronic contribution and the local structure perturbations on the host VO_2_ upon W^6+^-doping, which indicates that both contributions are responsible for the reduced T_C_^18^.

The detailed mechanisms involved in the increase of Tc by doping trivalent ions: Al^3+^, Cr^3+^ and Ga^3+^ in VO_2_ are complex to describe since more phases will be involved. Indeed a substitution of Al^3+^ or Cr^3+^ dopants for V^4+^ in VO_2_ gives rise to additional insulating phases: two specific monoclinic (M2 and M3) and a triclinic (T) phases, apart from the most common monoclinic (M1) phase^29^,^30^,^31^.

From the literatures, considering the atomic radius and the valence states of each dopant, it is clear that the regulation of Tc by doping ions is always associated with the lattice distortion and the carrier density change. The change in the carrier density of VO_2_ inevitably occurs when the dopant ion is not tetravalent, which means that a donor/acceptor-type doping (i.e., charge doping) of the VO_2_ band structure may occur. Nevertheless, which factor plays the critical role in the T_C_ behavior is still unclear.

In order to reveal the intrinsic mechanism for the regulation of T_C_ by ions-doping, it is mandatory to identify the roles of the lattice distortion and the charge doping caused by dopants, and which one plays the main role in this mechanism. To this purpose, considering that tetravalent ions-doping may rule out the carrier contribution of tetravalent dopant ions to neighboring vanadium ions, i.e., to minimize the charge doping effect, we choose Ti^4+^ ion as the dopant. In Ti^4+^-doped VO_2_ system, it is imperative to clarify the behavior of Ti^4+^ dopants and their influence on the host VO_2_ lattice. To address the above issues, the most suitable tool is the X-ray absorption fine structure (XAFS) spectroscopy, because of its specific element selectivity and the sensitivity to the local structure (2–5 Å) around the absorber atoms as well as the electronic structure^32^.

In this work, Ti_x_V_1-x_O_2_ nanopowders were prepared by a hydrothermal method with a subsequent Ar annealing treatment. We systematically explored the electronic and local geometric structure of both the Ti dopants and the host V atoms in Ti_x_V_1-x_O_2_ samples using XAFS spectroscopy at both Ti and V K-edges. A combined experimental and theoretical analysis of the mechanism of the regulation of Tc by Ti-doping was performed for the first time. In addition, a comparative analysis of different ion-doping systems was also performed to identify the critical factors in regulating the T_C_ in ions-doped VO_2_ systems.

## Results

### The influence of Ti^4+^-doping on phase transition properties of VO_2_

**Figure 2:**
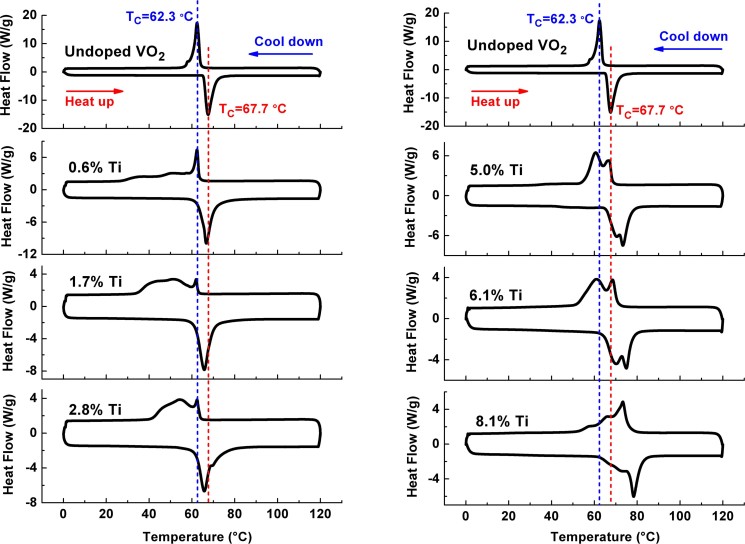
Comparison of DSC curves of Ti_x_V_1-x_O_2_ samples and an undoped VO_2_ (M1) sample during heating and cooling cycles.

Figure 2 shows the DSC curves of Ti_x_V_1-x_O_2_ samples with different Ti concentrations. At low Ti concentrations, as shown in Figure 2 (left), during the heating process the T_C_ slowly decreases with increasing Ti concentration. It reaches a minimum at the Ti concentration of 2.8%. During the cooling process the starting phase transition temperature is almost unchanged, and after the starting phase transition a broad exothermic peaks subsequently appear, indicating the occurrence of a non-uniform phase transition. At the higher Ti concentrations, e.g., 5.0%, 6.1%, and 8.1%, as shown in Figure 2 (right), during the heating process the T_C_ increases with increasing Ti concentration. The same trend of the T_C_ is also observed during the cooling process. In addition, the double endothermic/exothermic peaks appear during the heating/cooling process, probably due to the non-uniform doing or the polydispersity in the size distribution^19^.

Generally, the endothermic peaks appearing during the heating process are used to determinate the T_C_ of the ions-doped VO_2_. The T_C_ of the Ti_x_V_1-x_O_2_ samples slowly decreases reaching a minimum and then gradually increases with increasing Ti concentration, in agreement with data of Beteille *et al*.^22^. Previous researches did not show a unique behavior of the Ti^4+^-doping on regulating the T_C_. Most of researches reported the increase of T_C_, however, only showed the increase of T_C_ in high Ti concentration (>3%) due to weak ability of Ti dopants to regulate the T_C_^33^,^34^,^35^,^36^. In addition, it was found that the T_C_ saturated at around 80°C when the Ti doping concentration reaches to a higher level^35^. From the current results, it seems that the T_C_ decreases in initial low Ti concentration, while it increases gradually with further increasing Ti concentration in a certain concentration range.

Although Ti doping did not regulate the transition temperature significantly, Ti doping can effectively modify the thermochromic properties of VO_2_, such as the decrease of hysteresis sloop width of phase transition, the improvement of the temperature coefficients of resistance, and the enhancement of visible transmittance (T_vis_, 380–780 nm) and solar transmittance (T_sol_, 240–2600 nm)^33^,^34^,^35^,^36^,^37^. Compared with Ti_x_V_1-x_O_2_ film, there are no lattice mismatch and thermal stress for Ti_x_V_1-x_O_2_ nanopowders. Thus Ti_x_V_1-x_O_2_ nanopowders can be used to clarify the influence of Ti^4+^ dopants on the VO_2_ lattice structure, and then on the T_C_ of VO_2_.

### The influence of Ti-doping on the crystalline structure of VO_2_

**Figure 3:**
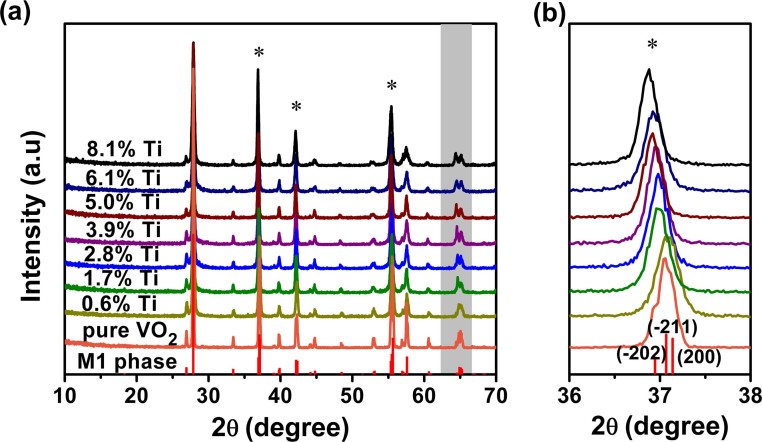
(a) XRD patterns measured at room temperature for the Ti_x_V_1-x_O_2_ samples with different Ti concentration. The peaks indicated with asterisk (*) clearly shift towards lower diffraction angles with increasing Ti concentration, such as a magnified view in panel (b).

Ion-doping inevitably modifies the host VO_2_ lattice structure, e.g., the phase transformation from a monoclinic to a rutile structure in the N_x_V_1-x_O_2_ systems (N = W^6+^, Mo^5+^ or Nb^5+^)^19^,^24^, and the formation of stabilized M2, M3, or T phases in the M_x_V_1-x_O_2_ systems (M = Al^3+^ or Cr^3+^)^29^,^31^. Figure 3 shows XRD patterns of Ti_x_V_1-x_O_2_ samples with different Ti concentration. No characteristic peaks of titanium oxides are observed, indicating the formation of titanium ions solid solutions with the VO_2_. At low Ti concentrations, the XRD patterns of the Ti_x_V_1-x_O_2_ samples (x = 0, 0.6%) match well with that of the monoclinic (M1, space group P2_1_/c) phase (JCPDS card No. 72-0514). With increasing Ti concentration, the diffraction peaks are consistent with that of the M1 phase except for two obvious peaks in the range 63.5° < 2θ < 66.0° (showed in the gray areas). In this range, the broad diffraction peak gradually splits into two obvious peaks with increasing Ti concentration. This scenario was also observed in the W_x_V_1-x_O_2_ system^18^,^26^. The appearance of the two peaks was associated to a rutile phase at high W concentration. Although the Ti-doping does not change the kind of crystal lattice of Ti_x_V_1-x_O_2_ samples, a local structure phase transition from the monoclinic to the rutile structure may occur in some regions of the samples with increasing Ti concentration. In addition, several diffraction peaks gradually shift to lower diffraction angles, e.g., the peaks in Figure 3b, pointing out upon Ti^4+^-doping a continuous increase of the interplanar spacing due to the larger radius of the Ti^4+^ ion (0.0605 nm) compared to the V^4+^ ion (0.058 nm). The opposite trend was observed when VO_2_ was doped with Al^3+^ ions (0.054 nm)^21^.

### The morphology and microstructure of Ti_x_V_1-x_O_2_ samples

**Figure 4:**
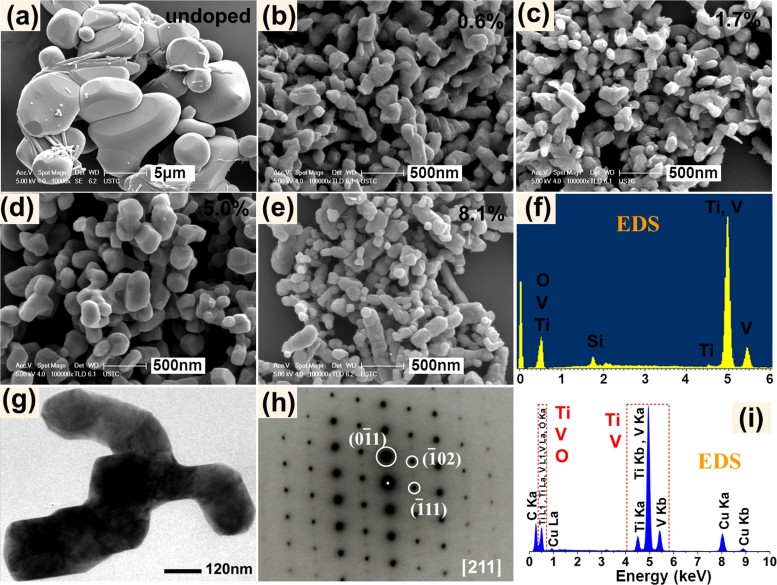
(a–e) SEM images of the Ti_x_V_1-x_O_2_ samples with different Ti concentration and an undoped VO_2_ sample; (f) EDS spectrum of the Ti_x_V_1-x_O_2_ sample (x = 0.6%); (g) TEM image of the Ti_x_V_1-x_O_2_ nanoparticles (x = 5.0%) and the corresponding selected area electron diffraction (SAED) pattern (h) and EDS spectrum (i).

Figure 4a–e show SEM images of Ti_x_V_1-x_O_2_ and undoped VO_2_ samples after the Ar annealing treatment. Ti_x_V_1-x_O_2_ samples are actually composed of nanoparticles (100–300 nm), compared with microparticles (1–10 μm) present in undoped VO_2_ sample. Moreover, the Ti doping had a strong effect on the morphological evolution of Ti_x_V_1-x_O_2_ samples. The Ti_x_V_1-x_O_2_ nanoparticles gradually reduce their mean particle size with increasing Ti concentration, due to the reduced crystallization ability (i.e., enhanced heterogeneous nucleation process) of Ti_x_V_1-x_O_2_ nanoparticles by Ti doping^35^. The same situation occurred in other ions-doped systems^21^.

In Figure 4f, the Energy dispersive X-ray (EDX) fluorescence spectrum obtained from SEM analysis shows the Ti, V, O, and Si characteristic peaks. Actually, the Si peak appears also due to the scattering induced by Si substrate. In Figure 4i, the EDS spectrum obtained from TEM analysis shows no Si peak since a Cu grid was used for supporting the sample, and clearly shows Ti, V, O, and Cu peaks. The Cu peaks are clearly due to the scattering induced by the Cu grid. Therefore, both EDS spectra obtained from TEM and SEM analysis confirm that Ti_x_V_1-x_O_2_ nanoparticles contain only Ti, V, and O elements.

Figure 4g shows the TEM image of Ti_x_V_1-x_O_2_ nanoparticles (x = 5.0%). The corresponding SAED pattern (Figure 4h) is in agreement with the diffraction pattern along the [211] crystal axis of the M1 phase (JCPDS No. 72-0514), showing the monoclinic single-crystalline nature of the Ti_x_V_1-x_O_2_ nanoparticles. In addition, the measured interplanar spacings are 0.10–0.12 Å larger than theoretical values, due to the lattice expansion when Ti ions were incorporated into the VO_2_ lattice.

### XAFS analysis

To clarify the behavior of Ti dopants and their influence on the host VO_2_, the local structures of both Ti and V atoms in the Ti_x_V_1-x_O_2_ samples, as well as their chemical states, were systematically investigated by XAFS spectroscopy at Ti and V K-edges.

**Figure 5:**
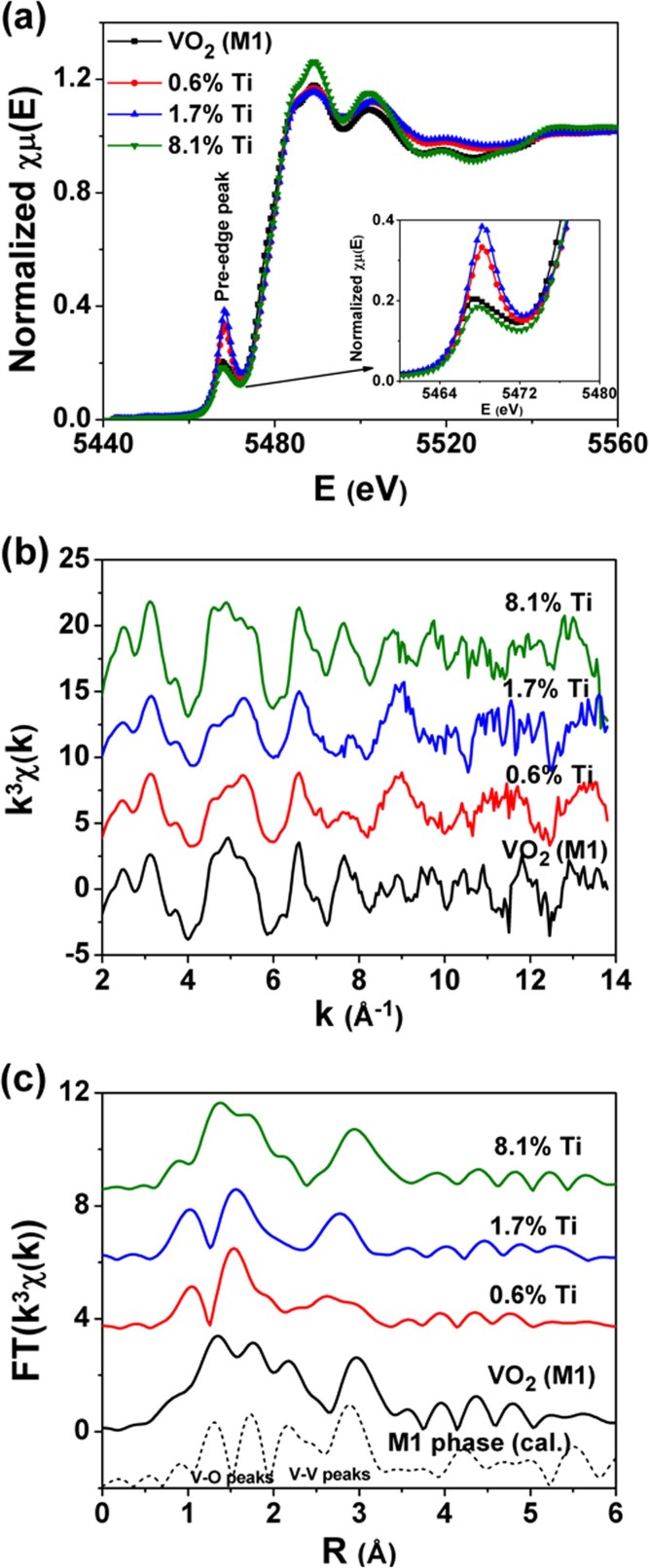
(a) V K-edge XANES spectra of the Ti_x_V_1-x_O_2_ samples (x = 0.6%, 1.7%, and 8.1%) and the undoped VO_2_ (M1) sample. The insert shows the enlarged view of the pre-edge absorption peak. (b) V K-edge EXAFS oscillations [k^3^χ(k)] and (c) their Fourier transforms (FTs), along with the theoretical curves of M1 phase for reference.

Figure 5a shows V K-edge XANES spectra of Ti_x_V_1-x_O_2_ and undoped VO_2_ (M1) samples. For vanadium oxides, the energy positions of the threshold, the pre-edge peak and the absorption-edge exhibit a monotonic dependence on the oxidation states of the absorber atoms according to Kunzl's law^26^,^38^,^39^. For Ti_x_V_1-x_O_2_ samples (x = 0.6%, 1.7%, 8.1%), the energy positions of the threshold, the pre-edge peak and the absorption-edges are almost the same and coincide with that of the undoped VO_2_ (M1), pointing out the tetravalent valence of V ions in the Ti_x_V_1-x_O_2_ samples. In addition, the pre-edge peak can be used to evaluate the changes in the local symmetry of V atoms, due to its sensitivity to the local coordination environment of the absorber atoms and the electron density of d states^38^,^39^,^40^. Pre-edge peak intensity increases with a lower local symmetry while decreases for a higher local symmetry of the absorber atoms. As an example, the pre-edge peak intensity is negligible in VO characterized by a regular octahedral symmetry (O_h_) around the absorber V atoms, while increases if the local symmetry is lower than the O_h_ symmetry such as in VO_2_, V_2_O_3_ and V_2_O_5_, reaching the maximum for vanadates with a tetrahedral coordination (Td)^38^. At low Ti concentrations (0.6% and 1.7%), Ti_x_V_1-x_O_2_ samples show an increased pre-edge peak intensity. However, a decreased intensity occurs at high Ti concentration, e.g., at 8.0% Ti concentration, and the pre-edge peak intensity decreases back to close to that of VO_2_ (M1). The change of the pre-edge peak intensity indicates that with increasing Ti concentration, the distortion of the VO_6_ octahedra around V atoms firstly increases to a maximum from the initial VO_6_ octahedra of VO_2_ (M1) and then decreases back to the VO_6_ octahedra of VO_2_ (M1).

V K-edge EXAFS data confirm the evolution of the local structure around V atoms. Figure 5b and c show V K-edge EXAFS oscillations [k^3^χ(k)] and their Fourier transforms (FTs), respectively. In Figure 5b, the 8.1% Ti sample shows similar EXAFS oscillations with the undoped VO_2_ (M1) sample, while 0.6% and 1.7% Ti samples show significantly different EXAFS oscillations respect to the VO_2_ (M1). Likewise, in Figure 5c, the FTs curves clearly demonstrate a similar local structure around V atoms for the 8.1% Ti sample and the VO_2_ (M1), in agreement with the theoretical spectrum of the VO_2_ (M1), i.e., their FTs curves exhibit the four characteristic peaks of M1 phase: the two peaks at ~1.33 and 1.75 Å´, corresponding to the first V–O coordination shell, and other two at ~2.15 and 2.95 Å´ associated to the V–V1_M_ and V–V_M_ shells. On the contrary, the low Ti concentration samples (0.6% and 1.7%) show FTs curves significantly different with that of VO_2_ (M1), compatible with local structures different from the standard M1 phase structure. Therefore, the local structure around V atoms deviates from the standard M1 phase structure first in the initial Ti doping process. With the further increase of the Ti concentration, the local structure around V atoms will return back to the M1 phase structure. This is accord with the change trend of VO_6_ octahedra in above V K-edge XANES analysis.

**Figure 6:**
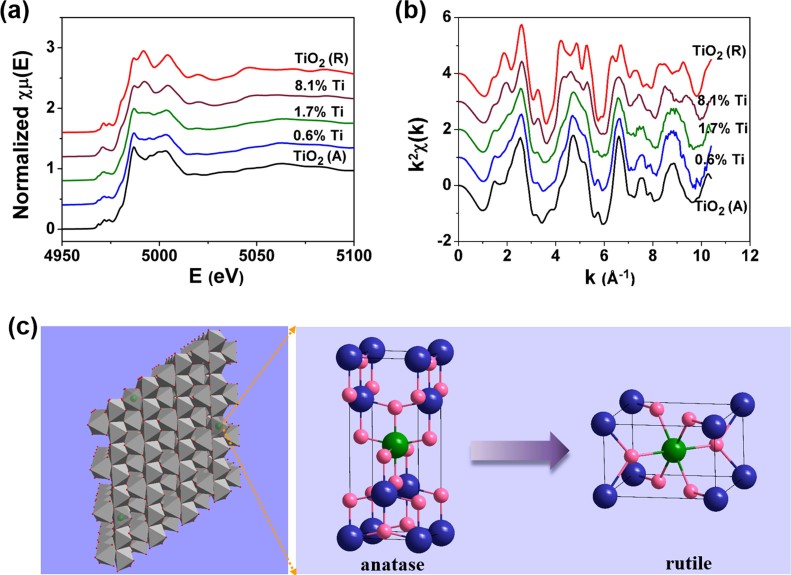
(a) Ti K-edge XANES spectra of the Ti_x_V_1-x_O_2_ samples (x = 0.6%, 1.7%, and 8.1%) and (b) their EXAFS oscillations [k^2^χ(k)], along with the reference samples of TiO_2_ (A) and TiO_2_ (R). (c) The local structure evolution of Ti dopants in the host monoclinic structure with increasing Ti concentration. The green balls in the panel denote the titanium atoms.

To understand the intrinsic local structure change around the host V atoms within the doping process, we then focus on the local structure around Ti dopants. Figure 6a and b show Ti K-edge XANES spectra and their EXAFS oscillations [k^2^χ(k)] of Ti_x_V_1-x_O_2_ samples, respectively, which both depict the evolution of the local structure around Ti dopants. The 0.6% and 1.7% Ti samples show similar XANES spectra with TiO_2_ (A) except for the pre-edge structure (Figure 6a). When the Ti concentration increases to 8.1%, the XANES spectrum matches well with that of TiO_2_ (R). This remarkable and systematic evolution also appears in the Ti K-edge EXAFS oscillations. As shown in Figure 6b, the 0.6% and the 1.7% Ti samples exhibit the characteristic EXAFS oscillations of TiO_2_ (A) while the 8.1% Ti sample exhibits the characteristic EXAFS oscillations of TiO_2_ (R). Therefore, Ti K-edge XANES and EXAFS data both indicate that the local structure around Ti dopants changes from the TiO_2_ (A)-like to the TiO_2_ (R)-like structure with increasing Ti concentration (see Figure 6c). Namely, at the initial low Ti concentration, the local anatase structure around Ti dopants is formed in the host monoclinic VO_2_ structure, and subsequently a local rutile structure around Ti dopants is gradually formed with increasing Ti concentration. The local structure dynamics of Ti dopants ought to be responsible for the local structure change of the host V atoms. Moreover, the local rutile structure around Ti dopants perfectly account for the appearance of two peaks in the range 63.5° < 2θ < 66.0° in the XRD patterns at high Ti concentrations. In addition, the energy positions of the absorption-edges of the Ti_x_V_1-x_O_2_ samples are constant and coincide with those of TiO_2_ (A) and TiO_2_ (R), pointing out the tetravalent valence of Ti ions in the Ti_x_V_1-x_O_2_ samples.

## Discussion

**Figure 7:**
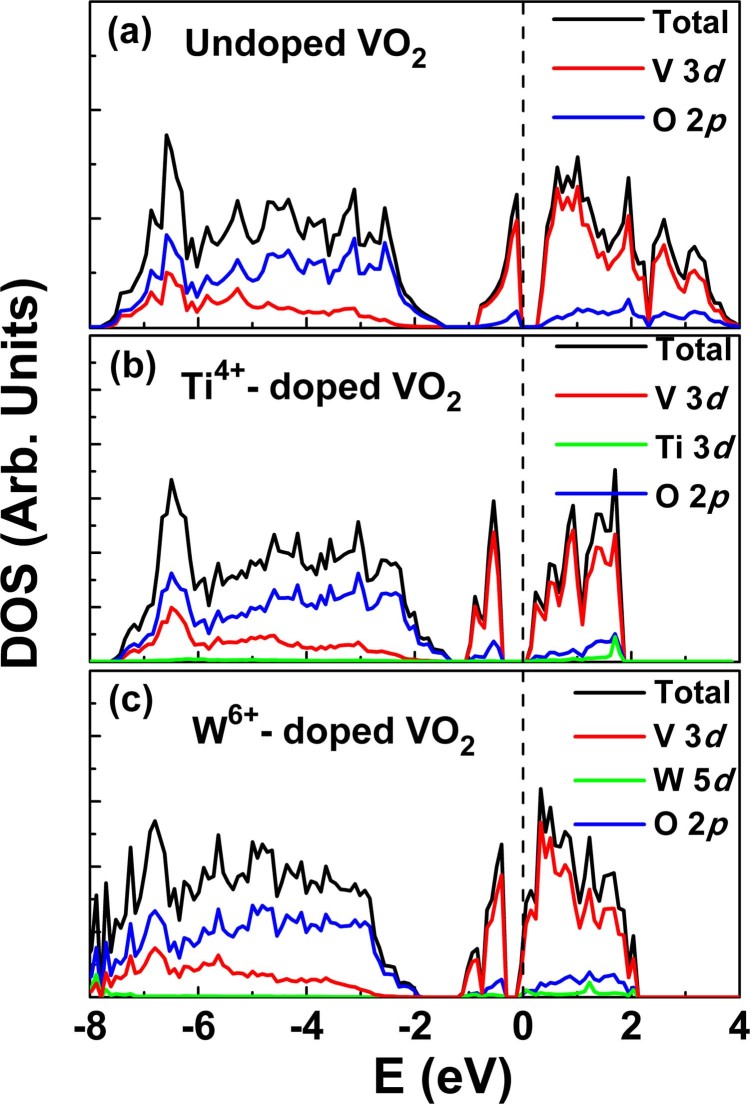
The DOS of (a) undoped VO_2_, (b) 6.25% Ti-doped VO_2_, and (c) 6.25% W-doped VO_2_ calculated by using the DFT method.

Based on our XAFS results, it seems that the doping VO_2_ with Ti^4+^ ions has a position between clearly donor- and acceptor-like defects, due to the same valence between the Ti^4+^ dopant and the V^4+^ ion. To confirm this scenario, we performed electron density of states (DOS) calculations using the projector augmented wave (PAW) method implemented in the Vienna *Ab-initio* Simulation Package (VASP)^41^. The PBE form of the generalized gradient approximation (GGA) and the DFT+U scheme^42^ (U = 4.0 and 6.6 eV for V and Ti atoms, respectively) were used to describe the electron exchange-correlation interaction. A 2 × 2 × 1 supercell with 48 atoms containing 15 V atoms, 32 O atoms and 1 Ti atom, corresponding to ~6.25% Ti concentration was used. For comparison, we also performed the similar DOS calculations of W-doped VO_2_, except U = 0 eV for W atom. Figure 7 compares the total and partial DOS of undoped VO_2_, 6.25% Ti-doped VO_2_, and 6.25% W-doped VO_2_. It can be observed that below the Fermi level, the total DOS and the partial V-3d/O-2p DOS do not show clear differences between the undoped VO_2_ and the 6.25% Ti-doped VO_2_ (Figure 7a and 7b), indicating the negligible influence of Ti^4+^ dopants on the valence band structure of VO_2_. But for W-doped VO_2_, the DOS (Figure 7c) shows the Fermi level in the bottom of the conduction band, indicating the electron doping of VO_2_ due to a charge transfer between W^6+^ and V^4+^ ions, in accord with the detection of reduction of V^4+^ to V^3+^ ions in W-doped VO_2_^18^,^26^. Therefore, from the above DOS calculations, we confirm that the incorporation of Ti^4+^ ions in VO_2_ basically do not induce a donor or acceptor doping of the VO_2_ band structure due to the lack of a charge transfer between Ti^4+^ and V^4+^ ions. This DOS calculations result can match with the constant valences of V and Ti ions from above XANES spectra, and the XPS spectra reported by Chen *et al*.^37^, which also showed the unchanged valence states of V^4+^ and Ti^4+^ in Ti-doped VO_2_. All of these indicate that the carrier concentration experience no significant change in Ti_x_V_1-x_O_2_ system. Accordingly, the charge doping effects caused by Ti^4+^ doping almost can be ruled out, and only the local structure perturbations can be considered to have the dominated effect on the regulation of Tc in Ti_x_V_1-x_O_2_ nanopowders samples.

Therefore, the two trend of the local structure change around V atoms induced by Ti-doping (from above V K-edge XAFS spectra), roughly corresponds to the two observed changes of the T_C_. On the basis of experimental and theoretical results, we consider that the mechanism of the regulation of Tc by Ti^4+^ doping is mainly associated with the local structure perturbations induced by Ti^4+^ dopants.

Our DSC results showed that the T_C_ slightly decreases in low Ti concentration level (within about 3% Ti concentration), while a small amount of W doping (within 3.4% W concentration) will result in a distinct Tc decreasing (Figure S1). Previous literatures also showed that the ability to regulate the T_C_ in the Ti_x_V_1-x_O_2_ system was smaller than the M_x_V_1-x_O_2_ (M = W^6+^, Mo^6+^or Nb^5+^) systems^12^,^19^,^26^,^33^,^36^,^43^. Table 1 shows the radius of several dopant ions and the V^4+^ ion. Ti^4+^ and W^6+^ ions have the radius close to each other, but W^6+^ ions have the considerably larger ability to regulate the T_C_ of VO_2_, i.e., a reduction in T_C_ by 20 ~ 30 K/at.% W for the bulk and by ~50 K/at.% W in nanostructures^11^,^24^,^43^,^44^.

In the case of W-doping, the VO_6_ octahedra shows a distortion trend with increasing W concentration, until the concentration of 1.7% (Figure S2). However, it cannot be suggested that the gradually decreasing of T_C_ (Figure S1) is mainly attributed to the distortion of VO_6_ octahedra since the electron doping of W^6+^ ions in VO_2_ is also conducive to the reduction of the T_C_^11^,^18^,^26^. In Ti_x_V_1-x_O_2_ system, the charge doping effects caused by Ti^4+^ doping can be ignored, i.e., decoupling the lattice distortion and charge doping effects on the phase transition behavior of VO_2_.

Due to the large ion size, when W or Ti atoms occupy the V sites, the substitution doping will yield the detwisting of the nearby monoclinic VO_2_ lattice in the similar way especially within low doping concentration. This type of lattice detwisting includes the decreasing V–V pairs tilting, depairing of dimerizated V–V pairs, and distorting the VO_6_ octahedra in surrounding monoclinic structure^18^,^24^,^28^. The distortion of VO_6_ octahedra induced by ions-doping can change the hybridization between V 3d and O 2p orbitals, resulting in the shift of π and π* bands near the Fermi level in the band structure of VO_2_, that finally changes the energy gap^24^.

The above comparative analysis suggested us that the charge doping (i.e., donor/acceptor doping) in VO_2_, plays a more fundamental role in the regulation of the T_C_, although the local structure perturbations induced by dopants has an inevitable influence on the T_C_. Actually, the smaller ability to regulate the T_C_ in the Ti_x_V_1-x_O_2_ system is most likely due to the negligible charge doping effect upon Ti^4+^-doping.

In conclusion, Ti_x_V_1-x_O_2_ nanopowders exhibit two trends for the T_C_: the T_C_ slightly decreases to a minimum and then increases with increasing Ti concentration. The behavior of Ti^4+^ dopants and their influence on the host VO_2_ lattice has been explored for the first time by XAFS spectroscopy and theory calculations. With increasing Ti concentration, the local structure around Ti dopants displays an evolution from a local anatase to a rutile-like structure, which induces a perturbation of the nearby monoclinic VO_2_ lattice. As a result, the distortion of the VO_6_ octahedra in the monoclinic VO_2_ lattice becomes more and more distinct at the initial doping stage. With the further Ti doping, the distorted VO_6_ octahedra will return to the initial VO_6_ octahedra together with the appearance of the local rutile structure around Ti dopants. The structure evolution induced by Ti doping is actually considered responsible for the observed trends of T_C_ in the DSC tests.

Finally, we should underline that, although the current Ti-doping research shown a direct influence of the local structure perturbations induced by Ti dopants on the regulation of T_C_, this modulation effect of the Ti doping strategy on VO_2_ materials is not such pronounced, in particular if we consider the variation of the phase transition temperature. By comparison of the ability to regulate the T_C_ in different ions-doping systems, such as W^6+^ dopants, we may claim that the charge doping for VO_2_ may play a critical role in the effective regulation of the T_C_ in no tetravalent ions-doped VO_2_ systems.

## Methods

### Synthesis of Ti_x_V_1-x_O_2_ nanopowders

The Ti-doped VO_2_ (Ti_x_V_1-x_O_2_) samples were synthesized by a hydrothermal method followed by an annealing treatment. The different amount of Ti (SO_4_)_2_ aqueous solution (0.01 M) were added to VO(acac)_2_ aqueous solutions under vigorous stirring. The value of x in Ti_x_V_1-x_O_2_ refers to the Ti atomic percent in the feed. Each of the final solutions was transferred to the Teflon cup, which was later heated in a sealed autoclave at 200°C for 24 hours. After the hydrothermal reaction, the precipitate was collected by centrifugation, washed with copious amounts of deionized water, N, N-dimethylformamide (DMF) and ethanol, and then dried in vacuum at 60° C. Finally, Ti_x_V_1-x_O_2_ samples were calcined under an argon (Ar) atmosphere at 700°C for 6 hours.

### Characterization

The crystalline structure of the Ti_x_V_1-x_O_2_ samples was determined by X-ray diffraction (XRD) using a theta/theta rotating anode X-ray Diffractometer (mode: TTR-III, Cu Kα radiation). The morphology and the microstructures were characterized with a Field-emission scanning electron microscope (FE-SEM, JEOL JSM-6700F) and transmission electron microscopy (TEM, JEM-2010(HR)). The phase transition behavior was studied by differential scanning calorimetry (DSC, Q2000).

### XAFS spectra measurement and analysis

XAFS spectra were measured at ambient temperature (~24°C) at the beamline 1W2B of the Beijing Synchrotron Radiation Facility (BSRF), using a Si(111) double crystal monochromator with an energy resolution (ΔE/E) of <1−3 × 10^−4^ @9 keV. Ti and V metal foils were respectively used for calibrating energy at the Ti K- and V K-edges. Ti K-edge XAFS spectra were collected in the fluorescence mode using a Lytle detector, while V K-edge XAFS spectra were collected in the transmission mode using ionization chambers filled with Ar/N_2_. V K- and Ti K-edges XAFS spectra were collected in the energy range of 5268–6251 and 4768–5419 eV, respectively. In the data processing procedure, the experimental absorption data were processed using the ATHENA module (version 0.8.054) implemented in the IFEFFIT package^46^,^47^.

## Author Contributions

Y.F.W. performed the experiments, collected and analyzed the data, and wrote the paper; L.L.F., S.C., F.H.C. and G.M.L. contributed to the TEM analysis and the interpretation of the results; Q.H.L. performed theoretical calculations; W.F.H. helped with XAFS analysis; Z.Y.W. and C.W.Z. initiated the topic, supervised the project and reviewed the paper.

## Supplementary Material

Supplementary Information Supplementary Information

## Figures and Tables

**Figure 1:**
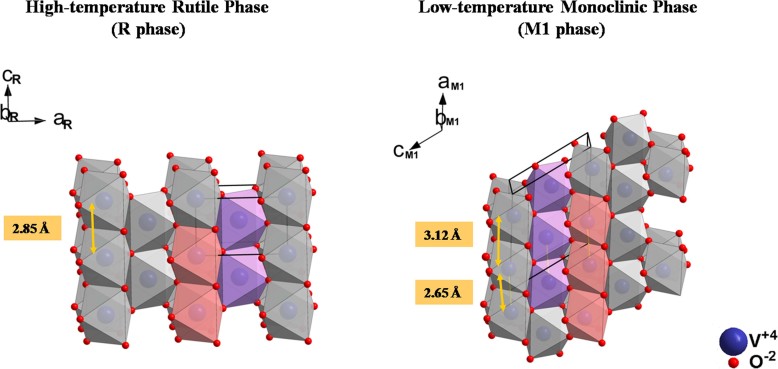
The crystal structures of low-temperature monoclinic (space group P2_1_/c) and high-temperature tetragonal rutile (space group P4_2_/mnm) phases of VO_2_.

**Table 1:** Comparison of the radius of different doped ions with V^4+^ ion

Element	Valence state	Ionic radius (pm)
V	+4	58
Ti	+4	60.5
Nb	+5	64
Mo	+6	59
W	+6	60

The ionic radius data was quoted from the literature of Shannon^45^.
