# Supplementary material for: Decoupling the Lattice Distortion and Charge Doping Effects on the Phase Transition Behavior of VO2 by Titanium (Ti4+) Doping
Source: Sci Rep. 2015 May 7;5:9328. doi: 10.1038/srep09328 (PMC4423444; doi:10.1038/srep09328)
Supplement: Supplementary Information [file srep09328-s1.pdf]

Supplementary Information

Decoupling the Lattice Distortion and Charge Doping Effects on the Phase Transition Behavior of VO_2 by Titanium (Ti^{4+}) Doping

Yanfei Wu[1], Lele Fan[2], Qinghua Liu[2], Shi Chen[2], Weifeng Huang[2], Feihu Chen[2], Guangming Liao[2], Chongwen Zou[2]* & Ziyu Wu[2,3]*

[1]*State Key Laboratory for Mesoscopic Physics, Department of Physics, Peking University, Beijing, 100871, People's Republic of China*

[2]*National Synchrotron Radiation Laboratory, University of Science and Technology of China, Hefei, 230029, People's Republic of China.*

[3]*Institute of High Energy Physics, Chinese Academy of Science, Beijing, 100049, People's Republic of China*

DSC curves of $W_xV_{1-x}O_2$ samples

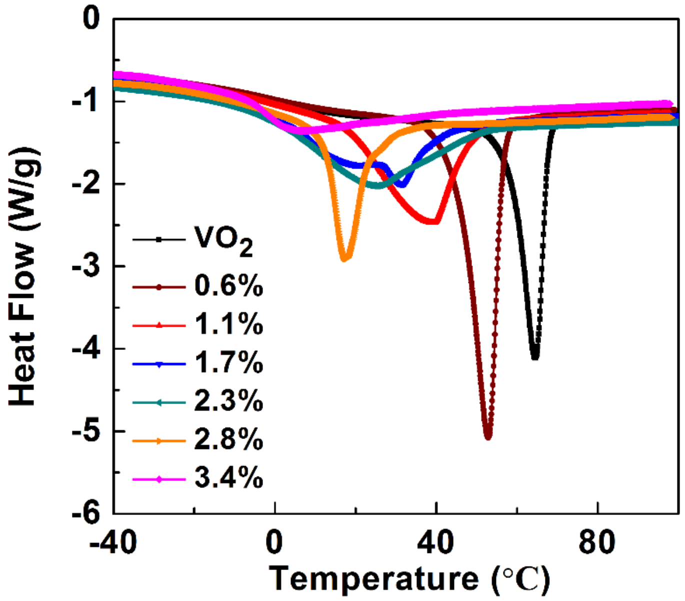

Figure S1. The DSC curves of $W_xV_{1-x}O_2$ samples with different W doping concentration during the heating cycles. The endothermic peak in each of the DSC curves is an indicator of T_C.

Figure S1 shows that the T_C is gradually reduced from 64.5 to 4.6 ℃ with increasing W concentration from 0% to 3.4%.

V K-edge XANES and EXAFS spectra of $W_xV_{1-x}O_2$ samples

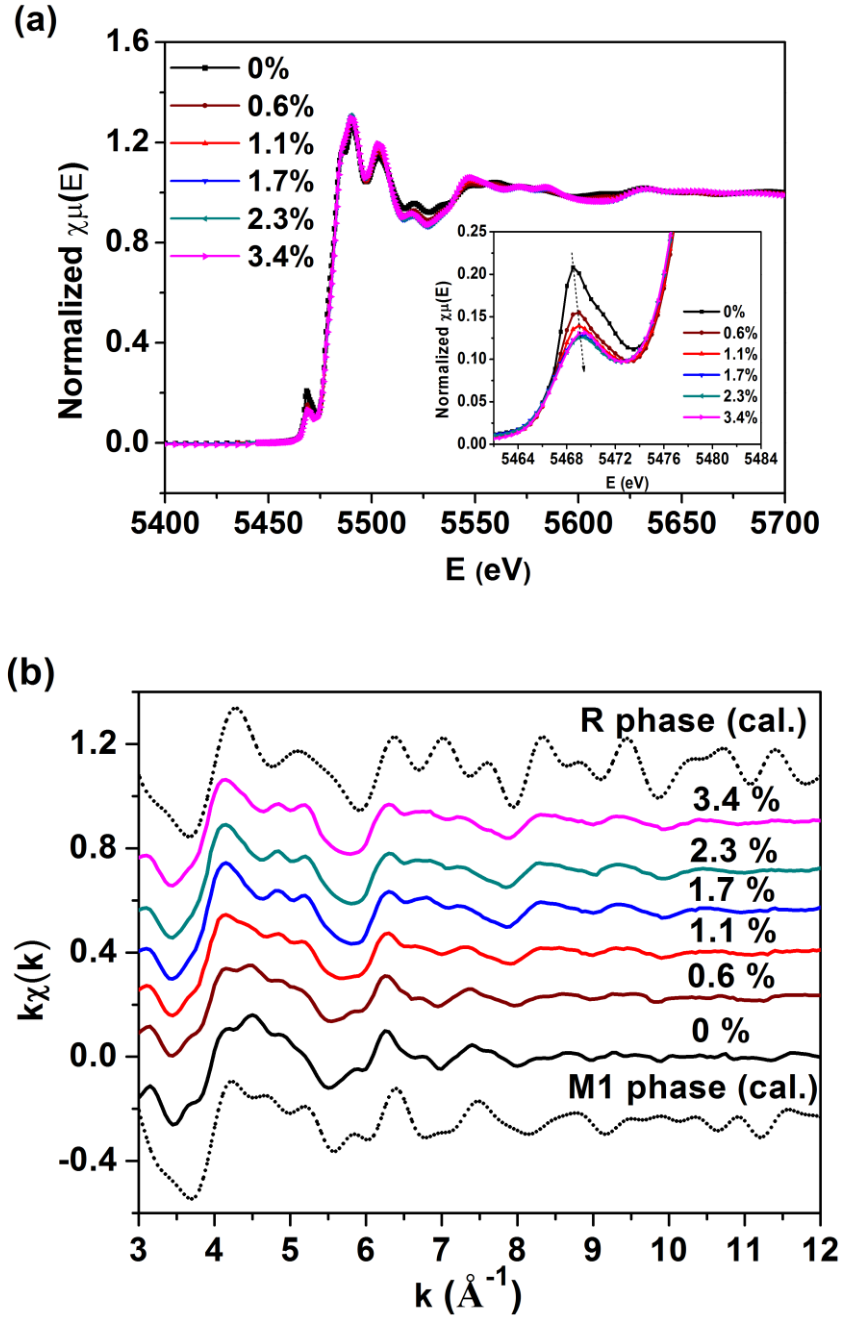

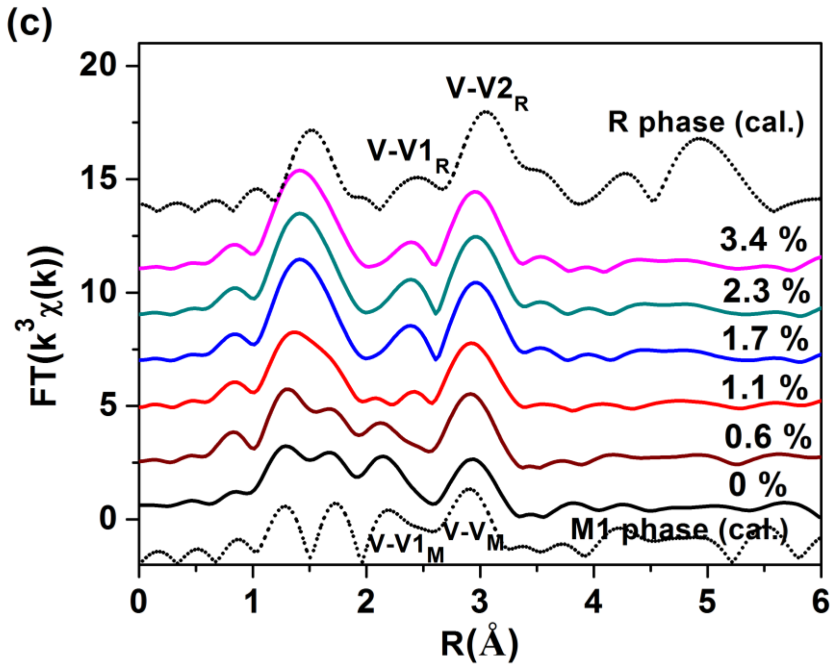

Figure S2. (a) V K-edge XANES spectra of $W_xV_{1-x}O_2$ samples with different W doping concentration. The insert shows a magnified view of the pre-edge peak. (b) V K-edge EXAFS oscillations kχ(k), and (c) their Fourier transforms (FTs), along with the calculated EXAFS data for VO_2 with monoclinic (M1, space group $P2_1/c$) and rutile (R, space group $P4_2/$ mnm) phases for references.

The pre-edge peak intensity is sensitive to symmetry variations and electronic configurations, thus it can be used to evaluate the variation in local symmetry of V atoms[1, 2, 3]. In the insert of Figure S2 (a), the intensity of the pre-edge peak decreases with increasing W doping, reflecting a higher octahedral symmetry around V atoms. That is the distortion of the VO_6 octahedra in $W_xV_{1-x}O_2$ samples gradually decreases, showing a distortion trend with increasing W concentration. And it seems that the distortion almost stop above the concentration of 1.7%.

Figure S2 (b) and (c) show the remarkable and systematic evolutions of the V K-edge EXAFS oscillations kχ(k) and their Fourier transforms for $W_xV_{1-x}O_2$ samples. The kχ(k) oscillations and their Fourier transform curves significantly change from M1 to R phases with increasing W concentration. Detailed analysis of the local structure evolutions around V atoms has be shown in our previous study [4]. When the W concentration increases to 1.7%, the local structure has completely transformed

into the rutile structure (R phase), as shown in Figure S2 (c), the two V–O split peaks merge in a single peak at ~1.44 Å, the V–V1$_M$ peak shifts to the V–V1$_R$ peak at 2.38 Å (two degenerate paths), and the V–V$_M$ peak turns to the V–V2$_R$ peak at 2.94 Å (eight degenerate paths). At the 1.1% doping sample, we observe the coexistence of M1 and R phases, on account of the presence of an asymmetric V–O peak at about 1.39 Å, and the co-occurrence of the V–V1$_M$ peak at 2.15 Å and the V–V1$_R$ peak at 2.38 Å.

W L$_3$-edge EXAFS spectra of $W_xV_{1-x}O_2$ samples

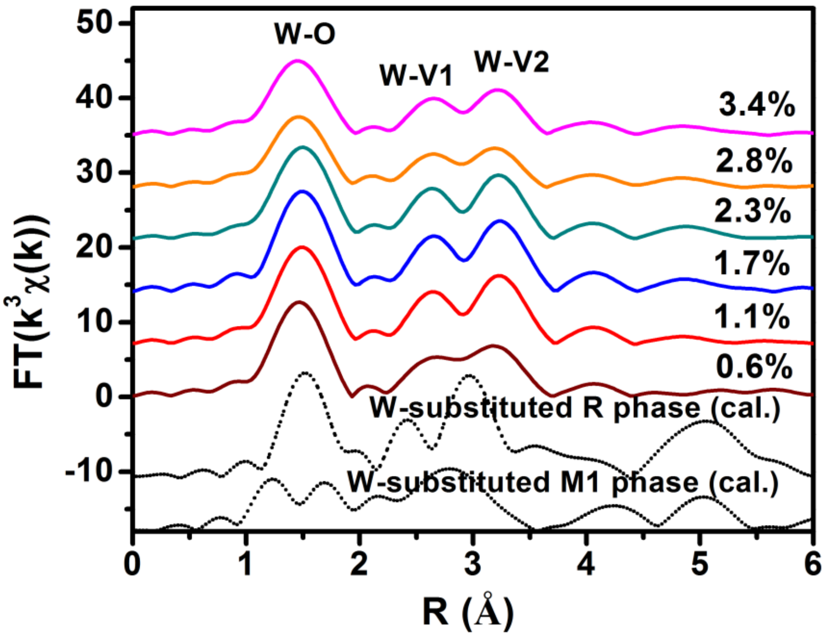

Figure S3 Fourier transforms (FTs) of W L$_3$-edge EXAFS oscillations for $W_xV_{1-x}O_2$ samples, along with the calculated EXAFS data for the W-substituted M1 and R phases for references.

In Figure S3, the FTs curves of $W_xV_{1-x}O_2$ samples are similar to the W–substituted R phase even in the low W concentration of 0.6%, showing the main features of the W–substituted R phase, while are different with the W–substituted M1 phase which has an obvious splitting for W–O peaks. For $W_xV_{1-x}O_2$ samples, the first three main peaks in the FTs curves correspond to W–O, W–V1, and W–V2 shells, respectively. In addition, the EXAFS curves of $W_xV_{1-x}O_2$ samples can be satisfactorily fitted with a tetragonal structural model that a W atom replaces the V

core in the R phase [4], reflecting the locally rutile structure around W dopants like the V atoms in the R phase of VO_2, even at low doping concentrations.

1. Wong, J., Lytle, F. W., Messmer, R. P. & Maylotte, D. H. K-EDGE ABSORPTION-SPECTRA OF SELECTED VANADIUM COMPOUNDS. *Phys. Rev. B* **30**, 5596-5610 (1984).
2. Giuli, G., Paris, E., Mungall, J., Romano, C. & Dingwell, D., V oxidation state and coordination number in silicate glasses by XAS. *Am. Mineral.* **89**, 1640-1646 (2004).
3. Chaurand, P. *et al.* New methodological approach for the vanadium K-edge X-ray absorption near-edge structure interpretation: Application to the speciation of vanadium in oxide phases from steel slag. *J. Phys. Chem. B* **111,** 5101-5110 (2007).
4. Wu, Y. *et al.* Depressed transition temperature of $W_xV_{1-x}O_2$: mechanistic insights from the X-ray absorption fine structure (XAFS) spectroscopy. *Phys. Chem. Chem. Phys.* **16,** 17705-17714 (2014).
